# Estimulação Cerebral Transcraniana não Farmacológica e não Invasiva para o Tratamento da Hipertensão Arterial

**DOI:** 10.36660/abc.20230851

**Published:** 2024-03-06

**Authors:** Juan Carlos Yugar-Toledo

**Affiliations:** 1 Faculdade de Medicina de São José do Rio Preto São José do Rio Preto SP Brasil Faculdade de Medicina de São José do Rio Preto - Medicina, São José do Rio Preto, SP - Brasil; 2 Instituto de Cardiologia e Endocrinologia Rio Preto Ltda São José do Rio Preto SP Brasil Instituto de Cardiologia e Endocrinologia Rio Preto Ltda, São José do Rio Preto, SP - Brasil

**Keywords:** Hipertensão, Estimulação Cerebral Transcaniana, Sistema Nervoso Autônomo

Os efeitos fisiológicos das técnicas de estimulação cerebral transcraniana nas células neuronais e sua associação com o complexo controle neural do sistema cardiovascular são razoavelmente bem compreendidos. A questão atual neste campo é se esta abordagem de tratamento é eficaz para a hipertensão arterial (HA). O método baseia-se na obtenção de efeitos terapêuticos através da passagem de uma corrente elétrica pelo cérebro. Este método de tratamento não farmacológico e não invasivo tem sido amplamente aplicado principalmente no tratamento de distúrbios psiconeurológicos^1^ e no controle da dor crônica.^2 - 4^ Consequentemente, a pesquisa científica concentrou-se principalmente na exploração de parâmetros de sinais elétricos com efeitos sedativos e analgésicos máximos. Atualmente, o escopo de aplicação de diversas modificações da eletroterapia transcraniana está se expandindo, sendo o método utilizado no tratamento abrangente de diversas doenças, incluindo a HA.^5^

A inervação cardíaca pelo sistema nervoso parassimpático e pelo sistema nervoso simpático (SNS) modula a frequência cardíaca (atividade cronotrópica) e a contração do músculo cardíaco (atividade inotrópica). A vasculatura periférica é controlada apenas pelo SNS, responsável pela resistência vascular periférica. Isso também medeia o reflexo barorreceptor, que, por sua vez, regula a pressão arterial (PA). A HA e o sistema nervoso autônomo (SNA) estão intimamente relacionados, de modo que distúrbios podem levar a comprometimentos vasomotores e diversas comorbidades, incluindo obesidade, HA, HA resistente e doença renal crônica. A disfunção autonômica também está associada a alterações funcionais e estruturais em órgãos-alvo (coração, cérebro, rins e vasos sanguíneos), aumentando o risco cardiovascular. A variabilidade da frequência cardíaca é um método de avaliação da modulação autonômica cardíaca para analisar o efeito de intervenções terapêuticas.^6^ Nesse contexto, a estimulação transcraniana por corrente contínua (ETCC) poderia ser uma ferramenta para melhorar a desregulação autonômica em pacientes com HA. Estudos recentes destacaram o papel potencial da terapia elétrica transcraniana no controle da pressão arterial.^5 , 7^ Foi estabelecido que a ETCC pode contribuir para ajustes agudos positivos no controle autonômico cardíaco e uma redução nos valores da PA de 24 horas em pacientes com HA refratária.^8 , 9^ Contudo, a questão da manutenção do controle da PA permanece em debate. A busca por parâmetros ótimos de corrente elétrica e a investigação de seus mecanismos de ação estão em andamento. Sabe-se que pacientes hipertensos apresentam maior tônus simpático cardíaco e vascular.^10^ Dada essa relação entre PA e atividade cortical, a ETCC parece ser uma ferramenta com bom potencial a ser explorada, já que os efeitos da estimulação cerebral invasiva (estimulação cerebral profunda e medula espinhal estimulação medular) já foram demonstrados com bons resultados no controle da PA.^11 , 12^

Como a desregulação central da PA está ligada a distúrbios do sono, a eletrossonoterapia pode ser um método baseado na aplicação de corrente pulsada contínua de baixa frequência (1-160 Hz) e baixa intensidade (até 10 mA) (principalmente de formato retangular) com duração de pulso curta (0,2-0,5 ms) para as estruturas do cérebro do paciente. Esta corrente afeta diretamente as formações estruturais do cérebro, propagando-se através do analisador visual nas regiões subcorticais e do tronco cerebral, bem como nos centros endócrinos que regulam as funções corporais. Segundo a literatura, o uso da eletrossonoterapia no tratamento da HA contribui para a normalização da regulação neuro-humoral do sistema cardiovascular, reduz o tônus hipersimpático e otimiza a hemodinâmica central através de sua influência nos centros subcorticais e no eixo hipotálamo-pituitário do SNA. Considerando o baixo custo e os efeitos colaterais leves, parece razoável propor este método para HA.

A relação entre os mecanismos fisiopatológicos da HA e o sistema nervoso central e periférico dos mecanismos de controle e regulação da PA envolve diversas áreas do cérebro (córtex sensório-motor, pré-córtex-frontal medial e córtex insular), como a modulação da regulação autonômica, mecanismos vasomotores somáticos, variações na PA e outros. Embora a área de pesquisa em ETCC tenha avançado muito na última década, novos avanços devem ser feitos para que diferentes técnicas possam ser utilizadas em todo o seu potencial. Os mecanismos subjacentes à estimulação não são totalmente compreendidos, embora vários estudos tenham tentado caracterizá-la com várias teorias mecanicistas propostas. Mais trabalhos para caracterizar os efeitos de todos os tipos de estimulação são necessários para elucidar completamente seus mecanismos de ação. A variabilidade individual na resposta à estimulação é uma questão importante em qualquer técnica metodológica, mas é crucial na estimulação cerebral.

Recentemente, foi demonstrado que a ETCC normaliza os níveis hemodinâmicos, de PA e os indicadores de FE podem ser recomendados como um dos métodos de terapia anti-hipertensiva em pacientes.^13^ Ensaios clínicos, avanços na biotecnologia, dispositivos de miniaturização, desenvolvimento de software e sistemas sem fio podem tornar a técnica valiosa e fácil de usar para distúrbios cardiovasculares com desequilíbrio autonômico, como HA.
