# Supplementary material for: Estimulação Cerebral Transcraniana não Farmacológica e não Invasiva para o Tratamento da Hipertensão Arterial
Source: Arq Bras Cardiol. 2024 Mar 6;121(1):e20230851. [Article in Portuguese] doi: 10.36660/abc.20230851 (PMC11081192; doi:10.36660/abc.20230851)
Supplement: Translation

# Translation: Non-Pharmacologic and Noninvasive Transcranial Brain Stimulation for the Treatment of Arterial Hypertension

**Authors:** Juan Carlos Yugar-Toledo

**Affiliations:** 1 São José do Rio Preto SP Brazil Faculdade de Medicina de São José do Rio Preto - Medicina, São José do Rio Preto, SP - Brazil; 2 São José do Rio Preto SP Brazil Instituto de Cardiologia e Endocrinologia Rio Preto Ltda, São José do Rio Preto, SP - Brazil

**Keywords:** Hypertension, Transcranial Stimulation, Nervous System Diseases

The physiological effects of transcranial brain stimulation techniques on neuronal cells and their association with the complex neural control of the cardiovascular system are somewhat understood. The current question in this field is whether this treatment approach is effective for arterial hypertension (HTN). The method is based on achieving therapeutic effects by passing an electric current through the brain. This non-pharmacological and noninvasive treatment method has primarily been widely applied for the management of psychoneurological disorders^1^and chronic pain control.^2 - 4^Consequently, scientific research has primarily focused on exploring electric signal parameters with maximal sedative and analgesic effects. Currently, the scope of application for various modifications of transcranial electrical therapy is expanding, with the method being used in the comprehensive treatment of various diseases, including arterial HTN.^5^

Cardiac innervation by the parasympathetic nervous system and the sympathetic nervous system (SNS) modulates the heart rate (chronotropic activity) and the contraction of the cardiac muscle (inotropic activity). The peripheral vasculature is controlled only by the SNS, which is responsible for peripheral vascular resistance. This also mediates the baroreceptor reflex (BR), which, in turn, regulates blood pressure (BP). HTN and the autonomic nervous system (ANS) are closely related, such that derangements can lead to vasomotor impairments and several comorbidities, including obesity, HTN, resistant HTN, and chronic kidney disease. Autonomic dysfunction is also associated with functional and structural changes in target organs (heart, brain, kidneys, and blood vessels), increasing cardiovascular risk. Heart rate variability is a method of assessing cardiac autonomic modulation to evaluate the effect of therapeutic interventions.^6^In this context, transcranial direct current stimulation (TDCS) could be a tool to improve autonomic dysregulation in patients with arterial HTN. Recent studies have highlighted the potential role of transcranial electrical therapy in BP control.^5 , 7^It has been established that TDCS may contribute to positive acute adjustments in cardiac autonomic control and a reduction in 24-hour BP values in patients with refractory HTN.^8 , 9^However, the issue of maintaining BP control remains subject to debate. The search for optimal electric current parameters and the investigation of its mechanisms of action are ongoing. It is known that hypertensive patients have greater cardiac and vascular sympathetic tone.^10^Given this relationship between BP and cortical activity, TDCS seems to be a tool with good potential to be explored, as the effects of invasive brain stimulation (deep brain stimulation and spinal cord stimulation) have already been demonstrated with good results in BP control.^11 , 12^

As BP central dysregulation is linked to sleep disturbances, electric-sonotherapy may be a method based on the application of low-frequency (1-160 Hz), low-intensity (up to 10 mA) continuous pulse current (primarily of rectangular shape) with short pulse duration (0.2-0.5 ms) to the structures of the patient's brain. This current directly affects the structural formations of the brain by propagating through the visual analyzer into the subcortical and brainstem regions, as well as the endocrine centers regulating bodily functions. According to the literature, the use of electric-sonotherapy in the treatment of HTN contributes to the normalization of neurohumoral regulation of the cardiovascular system, reduces hypersympathetic tonus, and optimizes central hemodynamics through its influence on subcortical centers and the hypothalamic-pituitary axis of the ANS. Considering the low cost and mild side effects, it seems reasonable to propose this method for HTN.

The relationship between the pathophysiological mechanisms of HTN and the central and peripheral nervous system of BP control and regulation mechanisms involves various areas of the brain (sensory-motor cortex, pre-cortex-frontal medial, and the insular cortex) such as the modulation of the autonomic regulation, somatic vasomotor mechanisms, variations in BP and others. Although the area of TDCS research has advanced greatly in the last decade, new advances must be made so that different techniques can be used to their full potential. The mechanisms underlying stimulation are not fully understood, although several studies have attempted to characterize it with various proposed mechanistic theories. Further work to characterize the effects of all types of stimulation is needed to elucidate their mechanisms of action fully. Individual variability in response to stimulation is an important issue in any methodological technique, but it is crucial in brain stimulation.

Recently, it has been demonstrated that TDCS normalizes hemodynamic, BP levels, and EF indicators can be recommended as one of the methods of antihypertensive therapy in young patients.^13^However, more extensive clinical trials, biotechnology advances, miniaturizing devices, software developments, and wireless systems may make the technique valuable and easy to use for cardiovascular disorders with autonomic unbalance such as HTN.
